# Exact solutions to cable equations in branching neurons with tapering dendrites

**DOI:** 10.1186/s13408-020-0078-z

**Published:** 2020-01-28

**Authors:** Lu Yihe, Yulia Timofeeva

**Affiliations:** 1 School of Psychology, University of Nottingham, Nottingham, UK; 2 Department of Computer Science, University of Warwick, Coventry, UK; 3 Centre for Complexity Science, University of Warwick, Coventry, UK; 4 UCL Queen Square Institute of Neurology, University College London, London, UK

**Keywords:** Branching and tapering dendrites, Passive and quasi-active membranes, Green’s function in metric graphs, Sum-over-trips

## Abstract

Neurons are biological cells with uniquely complex dendritic morphologies that are not present in other cell types. Electrical signals in a neuron with branching dendrites can be studied by cable theory which provides a general mathematical modelling framework of spatio-temporal voltage dynamics. Typically such models need to be solved numerically unless the cell membrane is modelled either by passive or quasi-active dynamics, in which cases analytical solutions can be reduced to calculation of the Green’s function describing the fundamental input-output relationship in a given morphology. Such analytically tractable models often assume individual dendritic segments to be cylinders. However, it is known that dendritic segments in many types of neurons taper, i.e. their radii decline from proximal to distal ends. Here we consider a generalised form of cable theory which takes into account both branching and tapering structures of dendritic trees. We demonstrate that analytical solutions can be found in compact algebraic forms in an arbitrary branching neuron with a class of tapering dendrites studied earlier in the context of single neuronal cables by Poznanski (Bull. Math. Biol. 53(3):457–467, 1991). We apply this extended framework to a number of simplified neuronal models and contrast their output dynamics in the presence of tapering versus cylindrical segments.

## Introduction

Most neurons share a common structure consisting of a soma, an axon and dendrites. Dendrites are typically the most extended parts. Their distinct morphologies started to be appreciated by many scientists from the 1890s through the exemplary drawings of Ramón y Cajal [32], and gradually the significance of dendritic functions in single cell neuronal computation became apparent (informative overviews from both an experimental and a theoretical perspective can be found in the series of books [10, 33, 37, 41]). A successful application of *cable theory* in modelling spatio-temporal voltage dynamics in dendritic arborisations can be attributed to Rall [31, 35]. The idea behind cable theory is to build models of dendritic voltage dynamics using the analogy of electrical circuits. The electrical circuits can be either passive (linear) or active (nonlinear), mimicking the absence or presence of voltage-gated ion channels in dendritic membranes. While the combination of realistic dendritic morphologies and active voltage-gated ion channels restricts such models to be solved by only numerical methods using a compartmental approach, mathematical analysis is possible when dendritic voltage dynamics can be described by a linearised (quasi-active) model. Although the linearised models ignore any active dynamics for spike generation, they provide the fundamental foundation for a better understanding of neuronal signal filtration and integration in single dendritic/axonal cables (e.g. [15, 26, 34]) and in more complex morphologies (e.g. [17, 18]).

Most models based on cable theory assume cylindrical dendritic segments, despite the fact that they are known to taper in many types of neurons, demonstrating initially rapid and then moderate decreases in dendritic radius from proximal to distal ends [3, 6, 20, 42]. Earlier mathematical modelling of dendritic voltage dynamics on continuous tapering structures was also attributed to Rall and co-authors with the focus on single tapering segments or dendritic trees that can be reduced to equivalent tapering cables [16, 31]. Later Poznanski [27] followed up the theoretical investigation of such tapering cables and identified the geometric types for continuous dendritic tapers that permit analytical solutions. In parallel, multiple studies focussed on finding analytical solutions in arbitrary branching neurons with cylindrical dendritic segments [2, 19, 22–25]). More recently, Glenn and Knisley [11] developed a method of obtaining analytical solutions in dendritic trees with continuous tapering structures and linearised voltage dynamics by solving a recursive transcendental equation, based on the original method designed by Major et al. [24, 25] for passive branching neurons with cylindrical dendritic segments.

In this work we use an alternative approach, the *sum-over-trips* framework, which was initially developed by Abbott et al. [2] from the path integral formulation. The original framework provided a method for calculating analytical Green’s functions (voltage response functions given a Dirac delta current injected at some discrete location) in arbitrary branching dendrites with cylindrical segments and passive membranes. Later this method was generalised to support quasi-active cell membranes [8] and electrically coupled neuronal networks [39]. Here we combine the results of [27] and [8], and introduce an extended theoretical framework for calculating Green’s functions in an arbitrary branching neuron with cylindrical or tapering segments and passive or quasi-active membranes. Moreover, we demonstrate that the set of geometric types identified in [27] makes it possible to describe the voltage dynamics in the entire branching structure by a unique Helmholtz equation, and thus allows us to apply the method of local point matching [43] for finding the Green’s functions in compact algebraic forms.

The paper is structured as follows. In Sect. 2 we introduce and study a model of a single tapering dendritic cable of infinite length. We demonstrate how this model can be reduced to a Helmholtz equation and can then be solved by calculating the corresponding Green’s function. Next, in Sect. 3 we consider an arbitrary branching neuron with tapering segments and quasi-active membranes, and extend the sum-over-trips framework for calculating Green’s functions in such neurons. In Sect. 4 we consider a number of illustrative examples and demonstrate the application of the extended sum-over-trips framework. In particular, we justify a consideration of a parabolic taper as one of the biologically realistic dendritic geometries, and then study (i) a soma and dendrite model and (ii) a ‘Y’-shaped dendritic tree model. Finally in Sect. 5 we provide a discussion of the further potential of this work. Some detailed mathematical derivations are collected in appendices for the interested reader.

## A mathematical model of a single tapering dendritic cable

### The generalised cable equation

We consider a single one-dimensional dendritic cable of an infinite length with its radius described by a smooth function $r(x)$, where $x\in\mathbb {R}$ denotes the spatial location along the cable. The cell membrane is modelled by an electrical circuit consisting of a passive part and an active part which mimics the dynamics of ion channels. Passive dynamics of the cell membrane are governed by the membrane capacitance $C_{m}$, the leak conductance $g_{l}$ and the resting membrane potential $E_{l}$. The ion current generated by an arbitrary type of ion channel can be modelled as $I_{\mathrm{ion}}=g_{\mathrm {ion}}(V)(V-E_{\mathrm{ion}})$, where $g_{\mathrm{ion}}(V)$ is the voltage-dependent conductance and $E_{\mathrm{ion}}$ is the ion’s reversal potential. According to the Hodgkin–Huxley formalism [14, 15] the form of $g_{\mathrm{ion}}(V)$ depends on the dynamics of the gating variables for a particular type of ion channel. The generalised cable equation with a single type of voltage-gated ion channel can then be written down as

1 $$ C_{m}\frac{\partial V}{\partial t}=-g_{l}(V-E_{l})-g_{\mathrm{ion}}(V) (V-E_{\mathrm{ion}})+\frac{1}{2R_{a} \rho(x)}\frac{\partial}{\partial x} \biggl[r^{2}(x) \frac{\partial V}{\partial x} \biggr]+I_{0}(x,t), $$

where the axial resistivity of the dendritic cytosol $R_{a}$ is a constant and

2 $$ \rho(x)=r(x)\sqrt{1+ \biggl( \frac{\textrm {d}r}{\textrm {d}x} \biggr)^{2}}. $$

The term

3 $$ I_{0}(x,t)=\frac{I_{\mathrm{inj}}(t)\delta(x-x_{0})}{2\pi \rho(x)} $$

models the input in the form of a current with a time course $I_{\mathrm {inj}}(t)$ injected at the location $x_{0}$. A detailed derivation of Eq. (1) is provided in Appendix 1.

It has been demonstrated earlier that the fully active (nonlinear) $I_{\mathrm{ion}}$ dynamics can be well approximated by a quasi-active (linearised) current $\widehat{I}_{\mathrm{ion}}$ [8, 18]. Equation (1) can thus be reduced to the following linearised system (from now on all membrane potentials are measured from rest):

4a $$\begin{aligned}& C_{m}\frac{\partial V}{\partial t}=-g_{l}V- \widehat{I}_{\mathrm{ion}}+\frac{1}{2R_{a} \rho(x)}\frac{\partial }{\partial x} \biggl[r^{2}(x)\frac{\partial V}{\partial x} \biggr]+I_{0}(x,t), \end{aligned}$$

4b $$\begin{aligned}& L_{\mathrm{ion}}\frac{\partial\widehat{I}_{\mathrm {ion}}}{\partial t}=-r_{\mathrm{ion}} \widehat{I}_{\mathrm{ion}}+V, \end{aligned}$$

where $r_{\mathrm{ion}}$ and $L_{\mathrm{ion}}$ are the effective resistance and inductance of the linearised ion channels respectively.

Next we will demonstrate how a set of transformations can convert the system of Eqs. (4a) and (4b) into an equivalent form that is ideal for the subsequent mathematical analysis. By defining

5 $$ \lambda(x)= \biggl[\frac{1}{2R_{a}g_{l}}\frac {r^{2}(x)}{\rho(x)} \biggr]^{1/2} $$

to be the characteristic length parameter [27, 30, 31], we can introduce a spatial scaling mapping

6 $$ X=\mu(x)= \int_{0}^{x}\frac{1}{\lambda(y)}\,\textrm {d}y, \quad x\in \mathbb {R}, $$

where $\mu:\mathbb {R}\to\mathbb {R}$ is bijective. Applying *μ* to Eqs. (4a) and (4b) leads to the following system of equations:

7a $$\begin{aligned}& \tau\frac{\partial V}{\partial t}=\frac{\partial^{2} V}{\partial X^{2}}-V+\frac{\lambda(x)}{r^{2}(x)} \frac{\textrm {d}}{\textrm {d}X} \biggl[ \frac{r^{2}(x)}{\lambda(x)} \biggr]\frac {\partial V}{\partial X}+ \frac{I_{0}(x,t)-\widehat{I}_{\mathrm{ion}}(x,t)}{g_{l}}, \end{aligned}$$

7b $$\begin{aligned}& L_{\mathrm{ion}}\frac{\partial\widehat{I}_{\mathrm {ion}}}{\partial t}=V-r_{\mathrm{ion}} \widehat{I}_{\mathrm{ion}}, \end{aligned}$$

where $\tau=C_{m}/g_{l}$ is the membrane time constant, and formally $x=\mu ^{-1}(X)$ for $\mu^{-1}$ the inverse mapping of *μ*. Similarly to the approach in [27] for the passive tapering cable, we define

8 $$ F(X)=F\bigl(\mu(x)\bigr)=\frac{r^{2}(x)}{\lambda(x)} $$

to be the geometric ratio, and reduce Eq. (7a) to the following form:

9 $$ \tau\frac{\partial V}{\partial t} =\frac{\partial^{2} V}{\partial X^{2}}-V+\xi(X) \frac{\partial V}{\partial X}+\frac{I_{0}(\mu^{-1}(X),t)-\widehat {I}_{\mathrm{ion}}(\mu^{-1}(X),t)}{g_{l}}, $$

where

10 $$ \xi(X)=\frac{1}{F(X)}\frac{\textrm {d}F}{\textrm {d}X}=\frac{\textrm {d}}{\textrm {d}X} \bigl[\ln F(X) \bigr]. $$

Introducing a voltage transformation

11 $$ V^{*}(X,t)=\mathcal {S}_{\phi}\bigl(V(X,t)\bigr)= \frac{V(X,t)}{\phi(X)}, $$

for

12 $$ \phi(X)= \biggl[\frac{F(0)}{F(X)} \biggr]^{\frac{1}{2}}, $$

we can further reduce Eq. (9) to

13 $$ \tau\frac{\partial V^{*}}{\partial t}=\frac{\partial^{2} V^{*}}{\partial X^{2}}-\beta(X)V^{*}+ \frac{I_{0}(\mu^{-1}(X),t)-\widehat{I}_{\mathrm{ion}}(\mu ^{-1}(X),t)}{g_{l}\phi(X)}, $$

where

14 $$ \beta(X)=1+\frac{\xi^{2}(X)}{4}+\frac{1}{2} \frac{\textrm {d}\xi}{ \textrm {d}X}. $$

Note $\mathcal {S}_{\phi}: \mathbb {R} \to\mathbb {R}$ is a bijective mapping, because $\phi(X)$ is always a positive constant given any *X* except the case when $r(x)=0$. Applying the voltage transformation $\mathcal {S}_{\phi}$ to Eq. (7b) gives us

15 $$ L_{\mathrm{ion}}\frac{\partial\widehat{I}_{\mathrm {ion}}}{\partial t}=-r_{\mathrm{ion}} \widehat{I}_{\mathrm{ion}}+V^{*}(X,t)\phi(X). $$

Therefore the above steps convert the model (4a) and (4b) defined in the $(x,t;V)$-coordinate into the following model in the $(X,t;V^{*})$-coordinate:

16a $$\begin{aligned}& \tau\frac{\partial V^{*}}{\partial t}=\frac{\partial^{2} V^{*}}{\partial X^{2}}-\beta(X)V^{*}+\frac{I_{0}(\mu^{-1}(X),t)-\widehat{I}_{\mathrm {ion}}}{g_{l}\phi(X)}, \end{aligned}$$

16b $$\begin{aligned}& L_{\mathrm{ion}}\frac{\partial\widehat{I}_{\mathrm{ion}}}{\partial t}=-r_{\mathrm{ion}}\widehat{I}_{\mathrm{ion}}+V^{*}(X,t) \phi(X). \end{aligned}$$

Since both the spatial and voltage mappings *μ* and $\mathcal {S}_{\phi}$ are bijective, the two models (4a) and (4b) and (16a) and (16b) are equivalent.

### The analytical solution

Applying the Laplace transform ($\mathcal {L}: f(t)\to f(\omega)$) to Eqs. (16a) and (16b) and assuming zero initial conditions we obtain

17a $$\begin{aligned}& \tau\omega V^{*}(X,\omega)=\frac{\partial^{2} V^{*}(X,\omega)}{\partial X^{2}}-\beta(X)V^{*}(X, \omega)+\frac{I_{0}(\mu^{-1}(X),\omega)-\widehat{I}_{\mathrm {ion}}(X,\omega)}{g_{l}\phi(X)}, \end{aligned}$$

17b $$\begin{aligned}& L_{\mathrm{ion}}\omega\widehat{I}_{\mathrm{ion}}(X, \omega)=-r_{\mathrm{ion}}\widehat{I}_{\mathrm{ion}}(X,\omega )+V^{*}(X,\omega) \phi(X), \end{aligned}$$

which can be reduced to the following compact form by simple substitution and rearrangement:

18 $$ \biggl[\gamma^{2}(X,\omega)-\frac{\partial ^{2}}{\partial X^{2}} \biggr] V^{*}(X,\omega)=\frac{I_{0}(\mu^{-1}(X),\omega)}{g_{l}\phi(X)}, $$

where

19 $$ \gamma(X,\omega)=\sqrt{\tau\omega+\beta(X)+\frac {1}{g_{l}(r_{\mathrm{ion}}+L_{\mathrm{ion}}\omega)}}. $$

**Figure 1:**
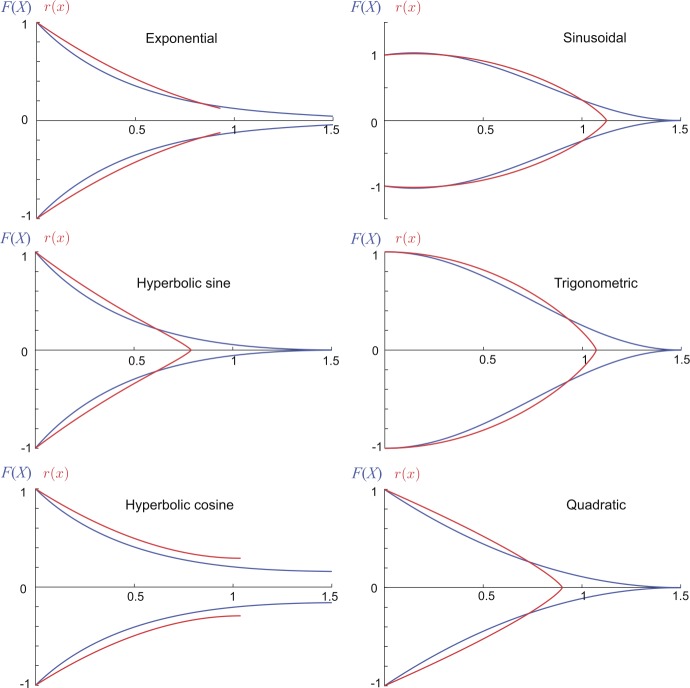
The six geometric types that permit analytical solutions in a single dendritic cable. The geometric ratios $F(X)$ (in blue) were computed as listed in Table 1 with $L=1.5$ and $\kappa=\pi/3$, except the Sinusoidal case with $L=0.15$ and $\kappa=\pi /2.7$. The dendritic radii (in red) were obtained numerically by solving Eq. (8) for $r(x)$. For plotting purposes, we assume $F(0)=r(0)=1$, and scale the *x*-coordinate by a factor of 10, where all coordinates and parameters are in arbitrary units

If $\gamma(X,\omega)$ is a constant in *X*, i.e. $\gamma(X,\omega )=\gamma(\omega)$ for all *X*, Eq. (18) can be rewritten as

20 $$ \bigl[\nabla^{2} - \gamma^{2}(\omega) \bigr] V^{*}(X,\omega) = -\frac{I_{0}(\mu^{-1}(X),\omega)}{g_{l}\phi(X)}, $$

where $\nabla^{2}$ is the Laplacian of $V^{*}$ on $X\in\mathbb {R}$. Note Eq. (20) has the form of a one-dimensional inhomogeneous Helmholtz equation with a complex-valued wavenumber $k = \gamma(\omega)\sqrt{-1}$, which is analytically solvable. Under the assumption that the parameters *τ*, $g_{l}$, $r_{\mathrm{ion}}$ and $L_{\mathrm{ion}}$ are constants, the special case when $\gamma(X,\omega )=\gamma(\omega)$ is equivalent to the case of $\beta(X)$ being simply a constant. One trivial scenario is when $r(x)$ is a constant and therefore $\beta(X)=1$, representing the case of a cylindrical cable. More generally, $\beta(X)$ being a constant reduces Eq. (14) to a Riccati equation, solutions to which provide six geometric types [27]. These six types are listed in Table 1, and their geometric ratios $F(X)$ and the corresponding dendritic radii $r(x)$ are illustrated in Fig. 1. Our modelling framework allows one to have an arbitrary choice for a spatial coordinate system and therefore to properly control the taper range associated with each type of dendritic taper.

**Table 1:** The six types of dendritic tapers that permit analytical solutions to a quasi-active cable equation. *κ*, *L* are positive constants and $n\in\mathbb {Z}$. Modified from [27]. Note that outside of the taper range, $F(X)$ increases

Type	*F*(*X*)	*β*(*X*)	Taper range
Exponential	exp(−2*κX*)	$1+\kappa^{2}$	$\mathbb {R}$
Hyperbolic sine	$\frac{\sinh^{2} \kappa(X-L)}{\sinh^{2} \kappa L}$	$1+\kappa^{2}$	(−∞,*L*]
Hyperbolic cosine	$\frac{\cosh^{2} \kappa(X-L)}{\cosh^{2} \kappa L}$	$1+\kappa^{2}$	(−∞,*L*]
Sinusoidal	$\frac{\cos^{2} \kappa(X-L)}{\cos^{2} \kappa L}$	$1-\kappa ^{2}$	$[L+\frac{\pi}{\kappa}n,L+\frac{\pi}{\kappa}(n+\frac{1}{2})]$
Trigonometric	cos^2^*κX*	$1-\kappa^{2}$	$[\frac{\pi}{\kappa }n,\frac{\pi}{\kappa}(n+\frac{1}{2})]$
Quadratic	$(1-X/L)^{2}$	1	(−∞,0]

Assuming the dendritic cable belongs to one of the six geometric types, we can introduce an additional spatial scaling $\gamma:X\to\bar{x}$ defined by

21 $$ \bar{x}=\gamma(\omega)X, $$

which normalises Eq. (20) into

22 $$ \bigl(\nabla^{2} - 1\bigr)V^{*}(\bar{x},\omega) = -A( \bar{x},\omega), $$

where

23 $$ A(\bar{x},\omega)=\frac{I_{0}(\mu^{-1}(\bar{x}/\gamma(\omega )),\omega)}{g_{l}\gamma^{2}(\omega) \phi(\bar{x}/\gamma(\omega))}. $$

The Green’s function of Eq. (22) can be found as

24 $$ H_{\infty}(\bar{x}) = \frac{1}{2}\exp\biggl(\gamma( \omega) \biggl\vert \frac{\bar{x}}{\gamma(\omega)} \biggr\vert \biggr) = \textstyle\begin{cases} \frac{1}{2}\exp(-\bar{x}), &\text{if $x,X\geq0$,}\\ \frac{1}{2}\exp(\bar{x}), &\text{if $x,X< 0$,} \end{cases} $$

as Eq. (22) is a special case of the inhomogeneous Helmholtz equation without any boundary conditions, whose Green’s function is well known. The derivation of Eq. (24) can be found in Appendix 2. It allows us to find the general solution to Eq. (22) as

25 $$ V^{*}(\bar{x},\omega)= \int_{C} H_{\infty}(\bar{x},\bar{y})A(\bar{y},\omega) \,\textrm {d}\bar{y}, $$

where $C\subset\mathbb {C}$ is a curve obtained by $\gamma:\mathbb {R}\to C$ given any fixed $\omega\in\mathbb {C}$. Hence, in the $(x,\omega;V)$-coordinate we have

26 $$ V(x,x_{0},\omega)=\frac{\varPhi(x_{0},x)}{z(x_{0},\omega )}H_{\infty}\biggl(\gamma(\omega) \biggl\vert \int_{x_{0}}^{x}\frac{1}{\lambda(y)}\,\textrm {d}y \biggr\vert \biggr)I_{\mathrm{inj}}(\omega), $$

where

27 $$\begin{aligned}& \varPhi(x_{0},x)=\frac{\phi(\mu(x))}{\phi(\mu(x_{0}))}, \end{aligned}$$

28 $$\begin{aligned}& z(x_{0},\omega)=\frac{\pi}{R_{a}}F\bigl(\mu(x_{0})\bigr) \gamma(\omega)=\frac{\gamma(\omega)}{\lambda(x_{0}) r_{a}(x_{0})}, \end{aligned}$$

29 $$\begin{aligned}& r_{a}(x_{0})=\frac{R_{a}}{\pi r^{2}(x_{0})}. \end{aligned}$$

Here we define $z(x_{0},\omega)$ to be the characteristic admittance at location $x_{0}$, while $z^{-1}(x_{0},\omega)$ and $r_{a}(x_{0})$ are more commonly referred as the characteristic impedance and the axial resistance respectively. If $I_{\mathrm{inj}}(t)=\delta(t)$, i.e. $I_{\mathrm{inj}}(\omega)=1$, Eq. (26) is reduced to the so-called transfer function of the model:

30 $$ G_{\infty}(x,x_{0},\omega)=\frac{\varPhi (x_{0},x)}{2z(x_{0},\omega)}\exp \biggl(-\gamma(\omega) \biggl\vert \int_{x_{0}}^{x}\frac{1}{\lambda(y)}\,\textrm {d}y \biggr\vert \biggr), $$

which is also known as the transfer impedance [18].

Applying the inverse Laplace transform ($\mathcal {L}^{-1}:f(\omega)\to f(t)$) to Eq. (26), we obtain the solution in the time domain as

31 $$ V(x,x_{0},t)=\mathcal {L}^{-1}\bigl\{ V(x,x_{0},\omega)\bigr\} (t)= \int_{0}^{t} G_{\infty}(x,x_{0},t-s)I_{\mathrm{inj}}(s) \,\textrm {d}s, $$

where $G_{\infty}(x,x_{0},t)$ is the inverse Laplace transform of the Green’s function $G_{\infty}(x,x_{0},\omega)$ given in (30). In the limit $r_{\mathrm{ion}}\to\infty$, the system is reduced to a purely passive model with the Green’s function

32 $$ G_{\infty}(x,x_{0},t)=\frac{\varPhi(x_{0},x)\lambda (x_{0})r_{a}(x_{0})}{\sqrt{4\pi\tau t}}\exp \biggl(-\beta\bigl(\mu(x)\bigr) \frac{t}{\tau}-\frac{\tau}{4t} \biggl[ \int_{x_{0}}^{x}\frac{1}{\lambda(y)}\,\textrm {d}y \biggr]^{2} \biggr). $$

**Figure 2:**
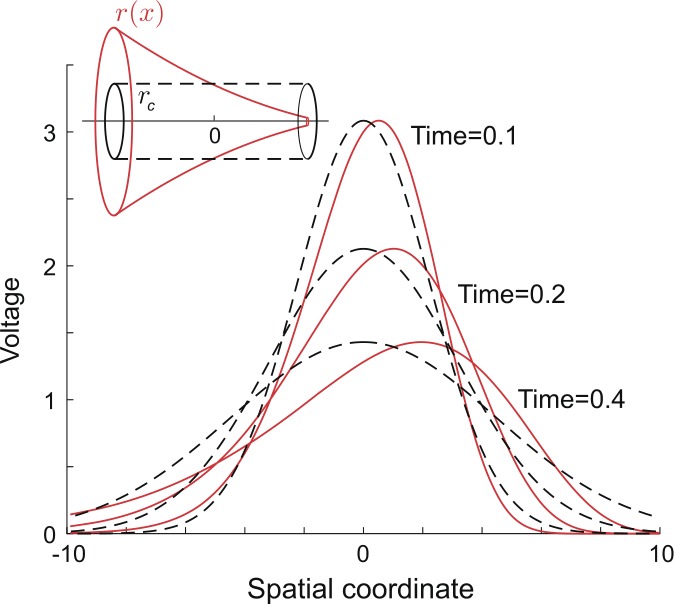
The Green’s function snapshots at three different time points in two (tapering and cylindrical) passive dendritic cables demonstrating the asymmetry in the spread of voltage in the tapering case (a slice of the tapering cable is illustrated in red and that of the cylindrical cable is in dashed black). All the coordinates and parameters are in arbitrary units: $2R_{a}g_{l}=1$, $R_{a}=4\pi^{3/2}$, $\tau=4$, radius of the cylindrical dendrite $r_{c}=1$, radius of the tapering cable at the origin $r(x=0)=1$. The tapering dendritic cable belongs to the exponential type with $\kappa=\pi/3$. The Dirac-delta input is placed at $x=0$

The system can be further reduced to the cylindrical case by considering $r(x)$ to be some constant $r_{c}$. Examples of the Green’s function profiles in the passive tapering and cylindrical dendritic cables are illustrated in Fig. 2, clearly demonstrating the asymmetry in the dispersion of the voltage in the tapering case.

## A mathematical model of a branching neuron with tapering dendrites

### The neuronal morphology

**Figure 3:**
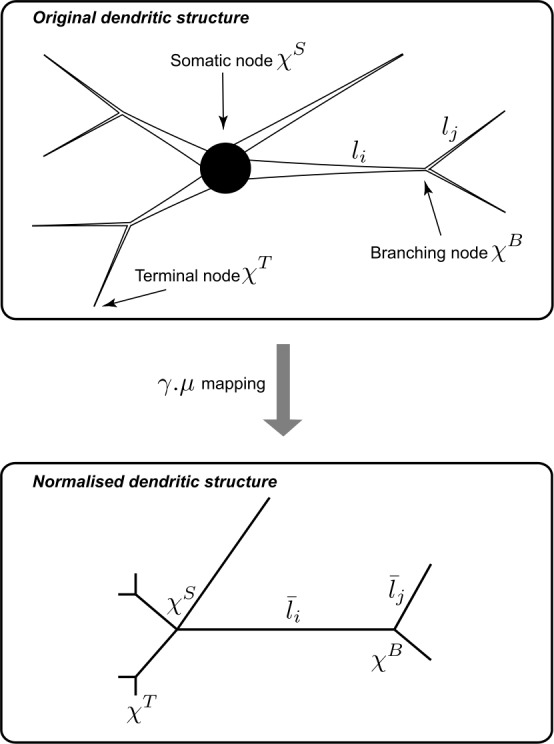
Top panel: A branching structure of a neuron with tapering dendrites modelled by a metric graph *Γ*. Bottom panel: A normalised graph structure $\varGamma^{*}$ after application of the normalisation mapping $\gamma\cdot\mu$. Note this graph normalisation allows one to choose the local spatial coordinates independently and arbitrary on each dendritic segment

Here we consider an arbitrary branching neuron as illustrated in Fig. 3. The global morphology is modelled as a graph $\varGamma=(N,S)$, where *N* is the set of somatic, branching and terminal nodes (vertices) and *S* is the set of dendritic segments (edges). In particular, *Γ* is a metric graph whose weighted edges are associated with the physical lengths $l_{i}$ for all dendritic segments $i\in S$.

Similar to the spatial transformations (6) and (21) for the case of a single tapering cable, we define the mappings $\mu_{i}:x\to X$ and $\gamma_{i}:X\to\bar{x}$ locally on each segment *i* as

33a $$\begin{aligned}& X=\mu_{i}(x)= \int_{0}^{x}\frac{1}{\lambda_{i}(y)}\,\textrm {d}y, \end{aligned}$$

33b $$\begin{aligned}& \bar{x}=\gamma_{i}(X)=\gamma_{i}(\omega)X, \end{aligned}$$

assuming that each tapering segment is either cylindrical or belongs to one of the six types listed in Table 1. For convenience we simplify the notations by defining the global spatial mapping $\mu:x\to X$ as the ensemble of $\mu_{i}$, and the global normalisation mapping $\gamma:X\to\bar{x}$ as the ensemble of $\gamma _{i}$ for all *i*. Hence, we define a normalised graph as

34 $$ \varGamma^{*}=\gamma\cdot\mu(\varGamma). $$

Although $\varGamma^{*}$ and *Γ* share the same graph structure (see Fig. 3), their edge weights are different; $\bar{l}_{i}=\gamma_{i}(\omega)\mu_{i}(l_{i})$ is the normalised length of segment *i* in $\varGamma^{*}$.

### The voltage dynamics

The voltage dynamics in the entire branching structure *Γ* with quasi-active membranes can be fully characterised by the dynamics $V_{i}(x,t)$ for $x\in(0,l_{i})$ on each dendritic segment $i\in S$ and the boundary conditions at all the nodes $\chi\in N$. In particular, $V_{i}(x,t)$ is governed by the following system of equations similar to Eqs. (4a) and (4b) for a single cable:

35a $$\begin{aligned}& C_{m,i}\frac{\partial V_{i}}{\partial t}=-g_{l,i}V_{i}- \widehat{I}_{{\mathrm{ion}},i}+\frac{1}{2R_{a,i} \rho_{i}(x)}\frac {\partial}{\partial x} \biggl[r_{i}^{2}(x)\frac{\partial V_{i}}{\partial x} \biggr]+I_{0,i}(x,t), \end{aligned}$$

35b $$\begin{aligned}& L_{{\mathrm{ion}},i}\frac{\partial\widehat{I}_{{\mathrm {ion}},i}}{\partial t}=-r_{{\mathrm{ion}},i}\widehat{I}_{{\mathrm{ion}},i}+V_{i}, \quad x\in(0,l_{i}), \end{aligned}$$

where the input

36 $$ I_{0,i}(x,t)=\frac{I_{\mathrm{inj}}(t)\delta (x-x_{0})}{2\pi\rho_{i}(x)}\delta_{ij}, $$

which, comparing to Eq. (3), has an extra term $\delta _{ij}$, a Kronecker delta (i.e. $\delta_{ij}=1$ if $i=j$ and 0 otherwise) denoting the input location on segment *j*.

Although each segment can have different individual parameters indicated by index *i*, all the equations can be normalised if we investigate the equivalent dynamics on $\varGamma^{*}$ instead of *Γ*. In particular, by introducing the spatial transformations $\gamma\cdot\mu$ in (33a) and (33b), a voltage transformation $\mathcal {S}_{\phi}$ similar to Eq. (11) and the Laplace transformation $\mathcal {L}$, we can reduce Eqs. (35a) and (35b) into the following equation defined on each segment *i*:

37 $$ \bigl(\nabla^{2} - 1\bigr)V_{i}^{*}(\bar{x}, \omega) = -A_{i}(\bar{x},\omega), $$

where

38 $$ A_{i}(\bar{x},\omega)=\frac{I_{0,i}(\mu_{i}^{-1}(\bar{x}/\gamma_{i}(\omega )),\omega)}{g_{l,i}\gamma_{i}^{2}(\omega) \phi_{i}(\bar{x}/\gamma_{i}(\omega))}. $$

Note

39 $$ \gamma_{i}(X,\omega)=\sqrt{\tau_{i}\omega+ \beta_{i}(X)+\frac{1}{g_{l,i}(r_{{\mathrm{ion}},i}+L_{{\mathrm {ion}},i}\omega)}}, $$

and all the other variables are in the same forms as in Sect. 2, but with index *i*.

Since Eq. (37) is in the same form as the normalised Helmholtz equation (22) for all $i\in S$, $(\nabla^{2} - 1)$ is a linear operator acting on all the segments and thus on the entire graph $\varGamma^{*}$.

### The boundary conditions

All the boundary conditions are assumed to be governed by two physical laws: continuity of potentials and conservation of currents. Here we consider three types of boundary conditions: terminal, branching and somatic nodes (see Fig. 3). Working on $\varGamma^{*}$ instead of *Γ* requires to transform these boundary conditions by $\gamma\cdot\mu$, $\mathcal {S}_{\phi}$ and $\mathcal {L}$, details of which are provided in Appendix 3.

#### Terminal node

A terminal node $\chi^{T}$ is either killed or sealed, and it can be described by one of the following boundary conditions:

40 $$ V\bigl(\chi^{T},t\bigr)=0, $$

or

41 $$ \frac{\partial V}{\partial x}\bigg|_{x=\chi^{T}}=0. $$

#### Branching node

For a branching node $\chi^{B}$ with *K* attached individual segments, the following two conditions are required:

42a $$\begin{aligned}& V_{i}\bigl(\chi^{B},t\bigr)=V_{j} \bigl(\chi^{B},t\bigr), \quad i,j \in\{1, 2, \ldots, K \}, \end{aligned}$$

42b $$\begin{aligned}& 0=\sum_{i=1}^{K} \frac{1}{r_{a,i}(\chi^{B})}\frac{\partial V_{i}}{\partial x}\bigg|_{x=\chi^{B}}, \end{aligned}$$

where $r_{a,i}(\chi^{B})=R_{a}/(\pi r_{i}^{2}(\chi^{B}))$.

#### Somatic node

A lumped soma $\chi^{S}$ can be treated as a special node connecting *K* segments, and the somatic membrane potential $V_{S}(t)$ can be described by a quasi-active model as

43a $$\begin{aligned}& V_{S}(t)=V_{i}\bigl(\chi^{S},t \bigr), \quad i\in\{1,2,\ldots,K\}, \end{aligned}$$

43b $$\begin{aligned}& C_{S}\frac{\textrm {d}V_{S}}{\textrm {d}t}=-g_{S} V_{S}-I_{S}+\sum_{i=1}^{K} \frac{1}{r_{a,i}(\chi^{S})}\frac{\partial V_{i}}{\partial x}\bigg|_{x=\chi^{S}}, \end{aligned}$$

43c $$\begin{aligned}& L_{S}\frac{\textrm {d}I_{S}}{\textrm {d}t}=-r_{S}I_{S}+V_{S}, \end{aligned}$$

where the constants $C_{S}$, $g_{S}$, $r_{S}$ and $L_{S}$ determine the linearised dynamics of the somatic membrane.

### The general solution

Let ${H}_{ij}(\bar{x},\bar{y})$ be the Green’s function for the operator $(\nabla^{2} - 1)$ on $\varGamma^{*}$. We can find the analytical solution to the system (37) as

44 $$ V^{*}_{i}(\bar{x},\omega)= \int_{C_{j}} {H}_{ij}(\bar{x},\bar{y})A_{j}( \bar{y},\omega)\,\textrm {d}\bar{y}, $$

where $C_{j}\subset\mathbb {C}$ is a curve obtained by $\gamma\cdot\mu :(0,l_{j})\to C_{j}$ given any fixed $\omega\in\mathbb {C}$. When $\varGamma^{*}$ is an infinite single cable, Eq. (44) is reduced to Eq. (25), as ${H}_{ij}(\bar {x},\bar{y})$ is replaced by $H_{\infty}(\bar{x},\bar{y})$. In general cases, we can use the sum-over-trips framework introduced earlier in [2, 8], which demonstrate that the Green’s function ${H}_{ij}(\bar{x},\bar{y})$ is closely linked to $H_{\infty}(\bar{x},\bar{y})$. In particular, it can be written down as

45 $$ {H}_{ij}(\bar{x},\bar{y})=\sum _{\mathrm{trip}}A_{\mathrm{trip}}(\omega)H_{\infty}\bigl( \bar{l}_{\mathrm{trip}}(x,y)\bigr), $$

where a trip on $\varGamma^{*}$ starting at *x* on segment *i* and terminating *y* on segment *j* is similar to a random walk, but with the restrictions of changing direction only at the nodes. Since the physical trip length ${l}_{\mathrm{trip}}(x,y)\geq0$, Eq. (24) for each trip is reduced to

46 $$ H_{\infty}\bigl(\bar{l}_{\mathrm{trip}}(x,y)\bigr)=\frac{1}{2}\exp \bigl(-\bar{l}_{\mathrm{trip}}(x,y)\bigr), $$

where $\bar{l}_{\mathrm{trip}}(x,y)=\gamma\cdot\mu({l}_{\mathrm {trip}}(x,y))$ is the normalised trip length. In addition, each trip weight (the summand in Eq. (45)) has to satisfy the boundary conditions at all the nodes along the trip, which is encoded in the trip coefficients $A_{\mathrm{trip}}(\omega)$. We will introduce in Sect. 3.5 the new rules for constructing $A_{\mathrm {trip}}(\omega)$. More details on the explanation and construction of trips and the sum-over-trips framework can be found in [2, 8].

Finally ${H}_{ij}(\bar{x},\bar{y})$ in Eq. (45) allows us to construct the Green’s function for $V_{i}(x,y,\omega)$ on *Γ* as

47 $$ G_{ij}(x,y,\omega)=\frac{\varPhi _{ji}(y,x)}{z_{j}(y,\omega)}\sum _{\mathrm{trip}}A_{\mathrm{trip}}(\omega)H_{\infty}\bigl( \bar{l}_{\mathrm{trip}}(x,y)\bigr), $$

and the voltage responses for an arbitrary applied current as

48 $$ V_{i}(x,y,\omega) = G_{ij}(x,y,\omega) I_{\mathrm{inj}}(\omega), $$

where

49 $$\begin{aligned}& \varPhi_{ji}(y,x)=\frac{\phi_{i}(\mu_{i}(x))}{\phi_{j}(\mu_{j}(y))}, \end{aligned}$$

50 $$\begin{aligned}& z_{j}(y,\omega)=\frac{\pi}{R_{a}}F_{j} \bigl(\mu(y)\bigr)\gamma_{j}(\omega)=\frac{\gamma_{j}(\omega)}{\lambda _{j}(y)r_{a,j}(y)}, \end{aligned}$$

51 $$\begin{aligned}& F_{j}\bigl(\mu(y)\bigr)=\frac{r_{j}^{2}(y)}{\lambda_{j}(y)}, \end{aligned}$$

52 $$\begin{aligned}& r_{a,j}(y)=\frac{R_{a}}{\pi r_{j}^{2}(y)}. \end{aligned}$$

From now on we assume for simplicity that the parameters describing the electrical properties of the cell membrane are identical for all dendritic segments and thus drop the subscript *i* for them, because our focus is on dendritic tapers of individual segments only. The above expressions are similar to those in the case of a single cable introduced in Sect. 2.2, but they are defined locally on each segment. From now on we use the term *Green’s function (in the frequency domain)* when referring to $G_{ij}(x,y,\omega )$ rather than ${H}_{ij}(\bar{x},\bar{y})$, unless stated otherwise.

### Rules for constructing trip coefficients

A trip coefficient $A_{\mathrm{trip}}(\omega)$ for any trip is calculated by firstly initialising its value to be 1, and then multiplying it by a node factor $\alpha_{nm}(\omega)$ every time when the trip travels from segment *n* to *m*. Node factors are essentially derived from, and thus encode complete information of, boundary conditions at individual nodes. A detailed derivation is given in Appendix 3. This derivation serves as a constructive proof that the function ${H}_{ij}(\bar{x},\bar{y})$ in Eq. (45) constructed using the appropriate node factors satisfies the required boundary conditions in *Γ*. Similar derivations for cylindrical dendrites can be found in [2, 8]. Below we list the node factors for three types of boundary conditions discussed in Sect. 3.3, omitting *ω* for compactness.

#### Terminal node

A trip has to reflect at a terminal node $\chi^{T}$. For a killed terminal, the node factors is

53 $$ \alpha_{mm}=-1. $$

For a sealed terminal,

54 $$ \alpha_{mm}=2p_{m}^{T}-1, $$

where

55 $$\begin{aligned}& p_{m}^{T}=\frac{z_{m}}{z^{*}_{m}}, \end{aligned}$$

56 $$\begin{aligned}& z_{m}^{*}=\frac{\gamma_{m}+\xi_{m}/2}{\lambda_{m}r_{a,m}}. \end{aligned}$$

The expression for $\xi_{m}$ has a similar form to Eq. (10) for an infinite single cable, but it is defined locally for each segment. Since each node factor is associated with the direction of the trip travelling away from the corresponding node, this direction is assumed to be positive orientation of the local spatial coordinate.

#### Branching node

At a branching node $\chi^{B}$ a trip can either reflect or pass through. If the trip reflects at $\chi^{B}$, the node factor is

57 $$ \alpha_{mm}=2p_{m}^{B}-1. $$

If the trip passes through $\chi^{B}$,

58 $$ \alpha_{nm}=2p_{m}^{B} \varPhi_{nm}^{(\chi^{B})}, $$

where

59 $$\begin{aligned}& p_{m}^{B}=\frac{z_{m}}{\sum_{m} z^{*}_{m}}, \end{aligned}$$

60 $$\begin{aligned}& \varPhi_{nm}^{(\chi^{B})}=\frac{\phi_{m}{(\chi^{B})}}{\phi_{n}{(\chi^{B})}}, \end{aligned}$$

are defined for segment *m* and *n* connected at $\chi^{B}$.

#### Somatic node

At a somatic node $\chi^{S}$, the node factors have the same expressions as the branching node factors, that is, for a reflective trip,

61 $$ \alpha_{mm}=2p_{m}^{S}-1, $$

and, for a transitive trip,

62 $$ \alpha_{nm}=2p_{m}^{S} \varPhi_{nm}^{(\chi^{S})}, $$

whereas

63 $$ p_{m}^{S}=\frac{z_{m}}{z_{S}+\sum_{m} z^{*}_{m}}, $$

and

64 $$ z_{S}(\omega)=C_{S}\omega+\frac{1}{R_{S}}+ \frac{1}{r_{S}+L_{S}\omega} $$

is the somatic admittance.

### The method of local point matching

In order to find the analytical Green’s function of the system (35a) and (35b) in *Γ*, we apply the extended sum-over-trips framework with the new node factors described above. Although the theoretical convergence of the infinite sum in the Green’s function (47) can be proved using a similar argument to [1], truncation of terms is always required for any numerical computation of Green’s functions in an arbitrary branching neuron [5]. Instead, the method of local point matching [43] can be used to find Green’s functions in compact algebraic forms. We refer the reader to [43] for a summary of the algorithmic steps of this method, which requires modifications for the case of dendritic tapers on the following three entities:

- the normalisation of the spatial coordinates;
- the voltage transformation;
- the node factors.

As a result, we can obtain the Green’s function (47) in the following form:

65 $$ G_{ij}(x,y,\omega)=\frac{\varPhi _{ji}(y,x)}{2z_{j}(y,\omega)}J_{y}, $$

where $J_{y}$ can be found by the method of local point matching [43]. We can then use it in Eq. (48) to calculate $V_{i}(x,y,\omega)$ and take the inverse Laplace transform to get $V_{i}(x,y,t)$.

## Some examples with parabolic dendritic taper

### Basic geometry of a parabolic dendritic segment

In the upcoming examples of simplified neuronal models, we assume that all dendritic segments are either cylindrical or parabolic in geometry. The choice of the parabolic taper is justified by a number of experimental observations [3, 6, 20, 42], indicating that the tapering slope is steeper at the start of the segment and is getting flatter towards the end. In particular, the radius of a parabolic dendritic segment satisfies

66 $$ r(x)=r_{0}(1-ax)^{2}, $$

for $x\in[0,l_{0}]$, as we choose the local *x*-coordinate for the segment so that $r_{0}=r(0)$, $r_{1}=r(l_{0})$ are the initial and terminal radii respectively, where $l_{0}$ is the length of the segment, and $a=(1-\sqrt{r_{1}/r_{0}})/l_{0}$ defines the slope of the parabola.

**Figure 4:**
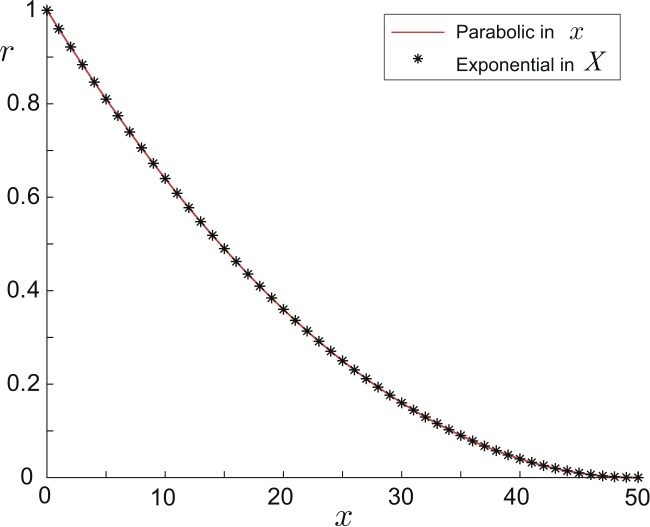
Comparison of the parabolic geometry given by Eq. (66) in red and the exponential type given by Eq. (67) in black asterisks, where $l=50$, $r_{0}=1$, $\lambda(0)=1$ are all in arbitrary units. $F(X)$ of the exponential type is transformed to $r(x)=r_{0}[F(X)]^{2/3}$. Their difference is at the order of 10^−16^

Under the assumption $[r'(x)]^{2}\ll1$, this parabolic taper can be well approximated by the exponential type (see [12] for the proof), whose geometric ratio can be found as

67 $$ F(X)=\frac{r_{0}^{2}}{\lambda(0)}e^{-2\kappa X}, $$

where $\lambda(x)$ and *X* are defined in Eqs. (5) and (6), and $\kappa=3a\lambda(0)/2$ is a positive constant characterising the slope of $F(X)$. Figure 4 justifies the fact that there are negligible difference between the two geometries. Therefore, we model all parabolic dendritic segments by the exponential type for its mathematical tractability demonstrated in Sect. 2. Combining Eqs. (8) and (67) we obtain

68 $$ X=\mu(x)=-\frac{3}{2\kappa}\ln(1-ax), $$

which defines the spatial mapping *μ* locally for each parabolic taper.

In addition we note that both hyperbolic sine and hyperbolic cosine types asymptotically approach the exponential type in the limit $l_{0}\to \infty$ (cf. Table 1), while the other three types have biologically unrealistic geometries since their $r(x)$ functions are concave (see Fig. 1). It has also been computationally validated in [9] and later mathematically proved in [4] that the parabolic taper is optimal in the transfer of current signals along a single dendritic cable.

### A soma and dendrite model: parabolic versus cylindrical cable

**Figure 5:**
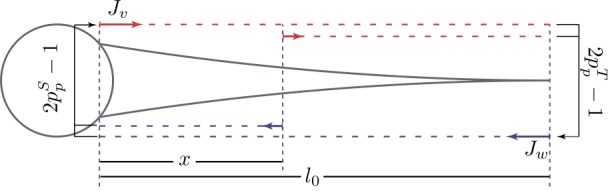
A neuronal model with a lumped soma and a single tapering dendritic branch. The coloured arrows denote the unknown variables $J_{v}$ and $J_{w}$ in Eqs. (74a) and (74b), while the black arrows represent the reflective node factors $2p_{p}^{S}-1$ and $2p_{p}^{T}-1$

Here we consider a simple model of a single dendritic cable with one end ($x=0$) attached to a lumped soma and the other end ($x=l_{0}$) being a sealed terminal node (see Fig. 5). Our earlier study of this model with a cylindrical cable of radius $r_{c}$ led to the the following Green’s function for the somatic response [43]:

69 $$ G_{c}(0,y,\omega)=\frac{p_{c}^{S} [\exp(-\gamma_{c}^{*} y )+\exp (\gamma_{c}^{*} (y-2 l_{0}) ) ]}{z_{c} [1-(2p_{c}^{S}-1)\exp(-2\gamma_{c}^{*} l_{0} ) ]}, $$

where

70 $$\begin{aligned}& p_{c}^{S}=\frac{z_{c}}{z_{c}+z_{S}},\quad z_{c}=\frac{\gamma_{c}}{\lambda_{c} r_{a,c}}=\frac{\gamma_{c}^{*}}{r_{a,c}}, \end{aligned}$$

71 $$\begin{aligned}& \gamma_{c}^{*}=\frac{\gamma_{c}}{\lambda_{c}},\quad\gamma_{c}= \sqrt{\tau\omega+1+\frac{1}{g_{l}(r_{\mathrm{ion}}+L_{\mathrm{ion}}\omega)}}, \end{aligned}$$

and $z_{S}$ is given in Eq. (64). The subscript *c* in the above expressions denotes the parameters in the cylindrical cable. The characteristic length parameter $\lambda_{c}$ and the axial resistance $r_{a,c}$ are simply constants. In the parabolic dendritic cable with radius described by Eq. (66), the geometry can be rewritten in the form of Eq. (67) with the spatial scaling *μ* given by Eq. (68), and then normalised by

72 $$ \gamma_{p}=\sqrt{\tau\omega+1+\kappa^{2}+ \frac{1}{g_{l}(r+L\omega)}}. $$

The subscript *p* here and below denotes the parameters in the parabolic cable. There are two node factors associated with this model: the reflective somatic node factor ($2p_{p}^{S}-1$) and the reflective terminal node factor ($2p_{p}^{T}-1$), where

73a $$\begin{aligned}& p_{p}^{S}=\frac{z_{p}(0)}{z_{p}(0)-\kappa+ z_{S}}, \end{aligned}$$

73b $$\begin{aligned}& p_{p}^{T}=\frac{z_{p}(l_{0})}{z_{p}(l_{0})+\kappa}. \end{aligned}$$

We can then apply the method of local point matching to construct the following linear system of equations (with the arrows in Fig. 5):

74a $$\begin{aligned}& J_{v}=\bigl[J_{w}h\bigl( \gamma_{p} \mu(l_{0})\bigr) + h\bigl(\gamma_{p} \mu(x)\bigr)\bigr] \bigl(2p_{p}^{S}-1\bigr), \end{aligned}$$

74b $$\begin{aligned}& J_{w}=\bigl[J_{v}h\bigl( \gamma_{p} \mu(l_{0})\bigr) + h\bigl(\gamma_{p} \mu(l_{0}-x)\bigr)\bigr] \bigl(2p_{p}^{T}-1\bigr), \end{aligned}$$

where $h(\bar{x})=\exp(-\bar{x})$. Solving the system (74a) and (74b), we obtain

75a $$\begin{aligned}& J_{v}=\frac{(2p_{p}^{S}-1) [ [(1-a(l_{0}-x))(1-al_{0}) ]^{3\gamma_{p}/2\kappa }(2p_{p}^{T}-1)+(1-ax)^{3\gamma_{p}/2\kappa} ]}{1-(2p_{p}^{S}-1)(2p_{p}^{T}-1)(1-al_{0})^{3\gamma_{p}/\kappa}}, \end{aligned}$$

75b $$\begin{aligned}& J_{w}=\frac{(2p_{p}^{T}-1) [ [(1-ax)(1-al_{0}) ]^{3\gamma_{p}/2\kappa }(2p_{p}^{S}-1)+(1-a(l_{0}-x))^{3\gamma_{p}/2\kappa} ]}{1-(2p_{p}^{S}-1)(2p_{p}^{T}-1)(1-al_{0})^{3\gamma_{p}/\kappa}}, \end{aligned}$$

which gives

76 $$ J_{y}=J_{v}h\bigl(\gamma_{p} \mu(y) \bigr)+J_{w}h\bigl(\gamma_{p} \mu(l_{0}-y) \bigr)+h\bigl(\gamma_{p} \mu\bigl( \vert x-y \vert \bigr)\bigr). $$

The Green’s function in the frequency domain can then be calculated using Eq. (65). In particular, the Green’s function for the somatic response can be found as

77 $$ \begin{gathered}G_{p}(0,y,\omega)=\frac{p_{p}^{S} [(1-ay)^{3\gamma_{p}/2\kappa }+(2p_{p}^{T}-1) [(1-al_{0})(1-a(l_{0}-y)) ]^{3\gamma_{p}/2\kappa} ](1-ay)^{3/2}}{z_{p}(y) [1-(2p_{p}^{S}-1)(2p_{p}^{T}-1)(1-al_{0})^{3\gamma_{p}/\kappa } ]}.\end{gathered} $$

For the comparison between the cylindrical and parabolic models, we focus only on their dendritic geometries and consider all other parameters to be the same. For the parabolic dendritic cable we assume $r_{0}=r_{c}$ and $r_{1}=0$. We can then rewrite the somatic Green’s functions (69) and (77) as

78 $$\begin{aligned} G_{c}(0,y,\omega)&=\frac{1}{z_{c}\tanh\gamma_{c}^{*} l_{0}+z_{S}} \frac{\cosh\gamma_{c}^{*}(l_{0}-y)}{\cosh\gamma_{c}^{*} l_{0}}, \end{aligned}$$

79 $$\begin{aligned} G_{p}(0,y,\omega)&=\frac{z_{p}(0)}{z_{p}(y)} \frac{1}{z_{p}(0)-\kappa+ z_{S}} \bigl[(1-ay) \bigr]^{3/2+3\gamma_{p}/2\kappa}. \end{aligned}$$

In the limiting case of a semi-infinite dendritic cable,

80 $$ \lim_{l_{0}\to\infty}G_{p}(0,y,\omega)=\lim _{l_{0}\to\infty} G_{c}(0,y,\omega)=\frac{2}{z_{c}+z_{S}}\exp \bigl(-\gamma_{c}^{*} y\bigr), $$

and in the limiting case of an infinitesimal dendrite,

81 $$ \lim_{l_{0}\to0}G_{p}(0,0,\omega)=\lim _{l_{0}\to0}G_{c}(0,0,\omega)=\frac{1}{z_{S}}. $$

Both limits can be derived analytically from Eqs. (78) and (79), but can also be heuristically obtained from a geometric perspective. In the first limit (80) the parabola asymptotically becomes a cylinder as $\kappa\to0$, while in the second limit (81) both models reduce to a single lumped soma with the somatic impedance $z_{S}^{-1}$.

**Figure 6:**
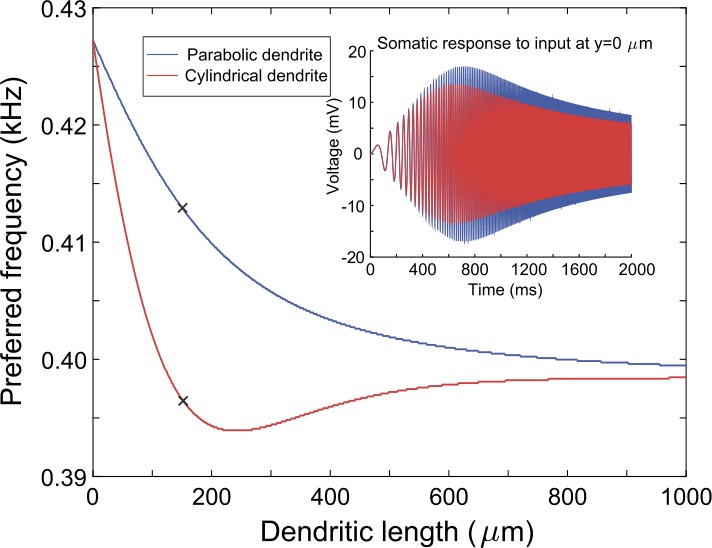
Comparison between the cylindrical (red) and parabolic (blue) single dendrite models. The preferred frequencies $\varOmega^{*}$ as functions of the dendritic length $l_{0}$ computed using the Green’s functions (78) and (79) with $x=y=0$. Insert: The time profiles of the somatic responses to a chirp input when $l_{0}=150 \mbox{ $\mu$m}$. The chirp current is defined to be $I_{\mathrm{chirp}}(t)=A_{\mathrm{chirp}} \sin(\omega_{\mathrm{chirp}} t^{2})$, where $\omega_{\mathrm{chirp}}=3\times10^{-4}\mbox{ kHz}$, $A_{\mathrm{chirp}}=0.2\mbox{ nA}$. The geometric parameters: $r_{c}=1 \mbox{ $\mu$m}$ for the cylindrical model, $r_{0}=1 \mbox{ $\mu$m}$ and $r_{1}=0 \mbox{ $\mu$m}$ for the parabolic model, $r_{S}=12.5 \mbox{ $\mu$m}$ for both models. The electrical parameters of the dendritic and somatic membranes are the same, and identical in both models: $C_{m}=1 \mbox{ $\mu$F $\cdot$ cm$^{-2}$}$, $g_{l}^{-1}=2000~\varOmega\cdot \mbox{cm}^{2}$, $R_{a}=100~\varOmega\cdot \mbox{cm}$, $r_{\mathrm{ion}}=1000~\varOmega \cdot \mbox{cm}^{2}$, $L_{\mathrm{ion}}=5\mbox{ H} \cdot \mbox{cm}^{2}$

To compare the two models in the presence of the quasi-active membranes, the preferred frequency $\varOmega^{*}$ can be introduced as the frequency at which the magnitude of the Green’s function is maximised. In Fig. 6 we plot the preferred frequencies for both models using the Green’s functions (78) and (79) with $x=y=0$. It is clear that the two curves are close to each other when the dendritic length $l_{0}$ is either extremely small or large, which means the two models behave similarly. This can be inferred from the two limits (80) and (81). We note that the curve for the parabolic model is monotonic while the curve for the cylindrical model is not. Within the dendritic length range investigated, the difference is maximised around $l_{0}=150\mbox{ $\mu$ m}$, with which the two models should behave the most differently; for this dendritic length, the time profiles of the two models’ somatic responses to a chirp current are illustrated in Fig. 6.

### A soma and dendrite model: parabolic versus compartmental cable

Here we still study the same parabolic model as in the previous section, but compare it to a compartmental model, consisting of an array of *N* cylindrical dendritic segments. We assume that these cylindrical segments share the same length $l_{0}/N$ while their radii are successively decreasing. Such compartmental models are commonly used in computational work (e.g. [9, 21, 42]) to approximate continuous tapering structures. In order to investigate the geometric effect on voltage amplitudes, we consider the two models to be purely passive, and set the dendritic geometries in such a way that the total membrane areas of the two models are equal, while assuming all the other parameters to be the same. In particular, we choose the dendritic radius of segment $i\in\{ 1, 2, \ldots, N\}$ in the compartmental model to be

82 $$ r_{c}(i)=\frac{r_{m}(i)+r_{M}(i)+\sqrt{r_{m}(i)r_{M}(i)}}{3}, $$

where

83a $$\begin{aligned}& r_{m}(i)=r \biggl(\frac{l_{0}}{N}(i-1) \biggr), \end{aligned}$$

83b $$\begin{aligned}& r_{M}(i)=r \biggl(\frac{l_{0}}{N}i \biggr), \end{aligned}$$

and the function $r(x)$ is defined by Eq. (66).

Since the two models are both passive, we only study the somatic responses at steady state (as $t\to\infty$) to a step current of strength $I_{\mathrm{step}}$ switched on at time $t_{0}$. Equivalently in the frequency domain, we have

84 $$ I_{\mathrm{inj}}(\omega)=\frac{I_{\mathrm{step}}}{\omega }e^{-t_{0}\omega}. $$

**Figure 7:**
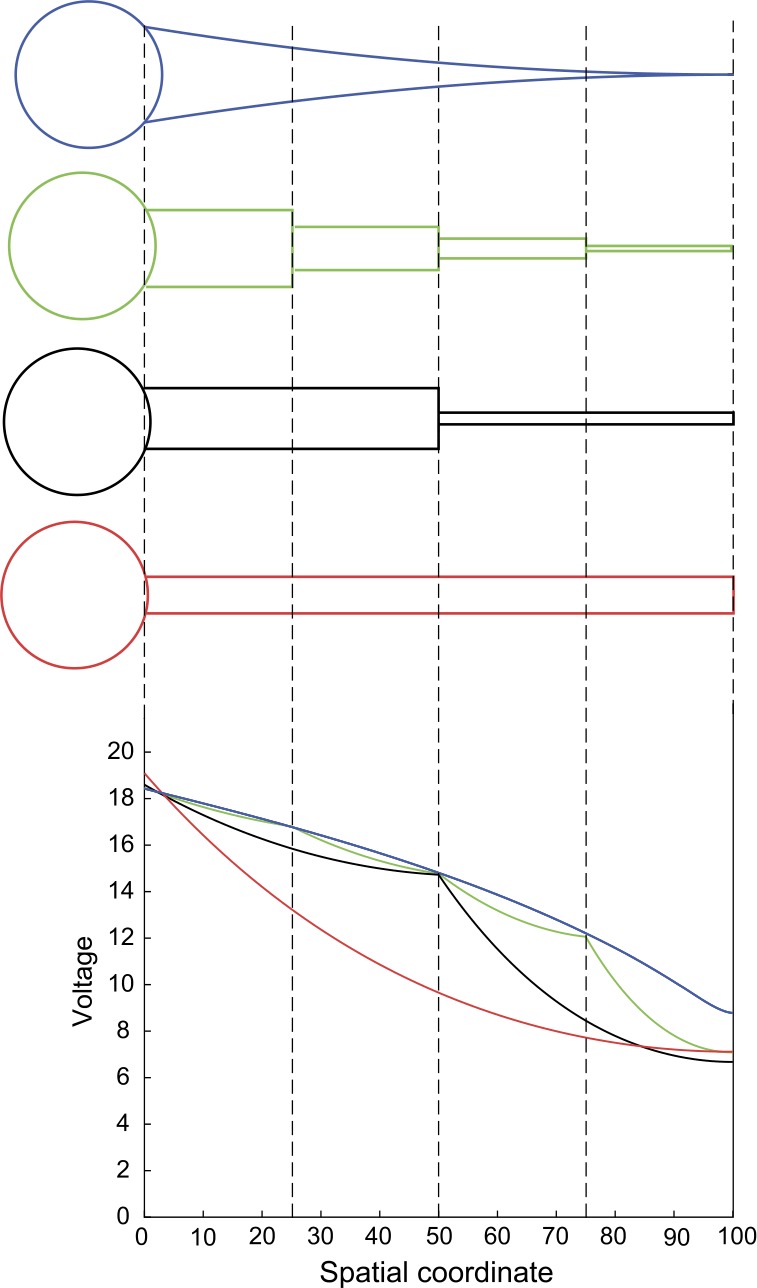
Somatic responses at steady state of the parabolic and compartmental models, when the step current given by (84) is placed at different input locations. Here the origin of the spatial coordinate is placed at the somatic node. The models are purely passive with the electrical parameters as in Fig. 6 except $R_{a}=1000~\varOmega \cdot \mbox{cm}$. The parabolic geometry is defined by Eq. (66), where $r_{0}=1~\mu\mbox{m}$, $r_{1}=0.01~\mu\mbox{m}$ and $l_{0}=100~\mu\mbox{m}$. The compartmental geometry is defined by Eq. (82) for $N=1, 2, 4$

Therefore, once we obtain the Green’s functions in the frequency domain, we can write down the voltage at steady state simply as

85 $$ V_{\mathrm{ss}}(x,y)=\lim_{t\to\infty}V(x,y,t)=I_{\mathrm{step}}G(x,y,0), $$

by applying the final value theorem for the Laplace transform. We can clearly see from Fig. 7 that the curve of $V_{\mathrm{ss}}(0,y)$ for the parabolic model is concave, while the curve for the compartmental model is convex on all individual segments. We checked that this property is always valid regardless of parameter choices. One could take Eqs. (78) and (79) as an example: when $\omega=0$, $G_{c}(0,y,0)$ is almost exponential in *y* while $G_{p}(0,y,0)$ follows a power law in *y* and the power $3/2+3\gamma _{p}/2\kappa>3$. This results in a large range of *y* that permits $V_{{\mathrm {ss}},p}(0,y)>V_{{\mathrm{ss}},c}(0,y)$.

We also verified that when *N* is very large (e.g. $N=1000$), the $V_{{\mathrm{ss}},c}(0,y)$ curve became indistinguishable from the $V_{{\mathrm{ss}},p}(0,y)$ curve in Fig. 7 (not illustrated here). However, considering computational expenses, a dendritic taper is usually approximated by a compartmental model with only a few segments ($N<10$ in [9, 21, 42]). Errors of such approximations are not negligible on segments whose radii are tiny (typically the segment near terminal), even when *N* is relatively large (e.g. $N=100$), because the input (characteristic) impedances are extremely different in the two models.

### A ‘Y’-shaped dendritic tree: parabolic versus cylindrical segments

**Figure 8:**
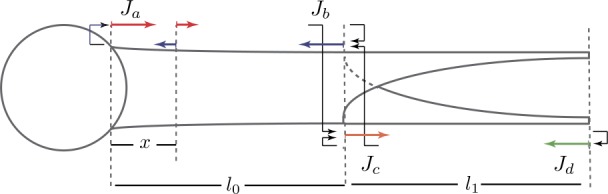
A neuronal model with a lumped soma and a ‘Y’-shaped dendritic tree. The coloured arrows denote the unknown variables in Eqs. (137a), (137b), (137c) and (137d), while the black arrows represent the node factors (see Appendix 4)

Here we consider a parabolic model of a passive neuron with a simplified ‘Y’-shaped dendritic tree. The dendritic tree consists of one cylindrical primary dendritic segment and two identical parabolic secondary segments that are attached to one another at the branching point ($x=0$). A lumped soma is attached to the other end of the primary segment ($x=-l_{0}$), and both secondary segments are sealed at the other ends ($x=l_{1}$), as illustrated in Fig. 8. We also consider a cylindrical model which differs from the parabolic model by only reducing the geometry of the secondary dendritic segments into cylinders using Eq. (82) with $N=1$.

**Figure 9:**
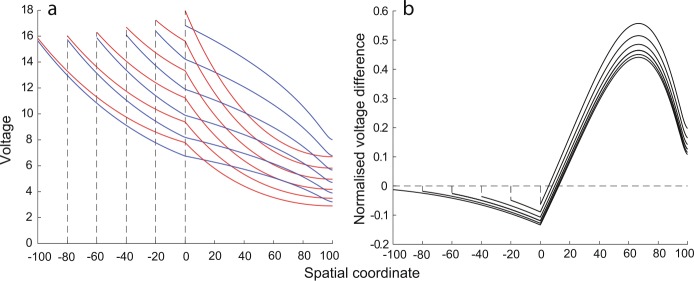
(**a**) Somatic responses at steady state of the cylindrical (red) and parabolic (blue) models to different input locations on the ‘Y’-shaped dendritic tree. Both models have a soma and a cylindrical primary dendrite of a radius $r_{c}=r_{0}=1~\mu\mbox{m}$ whose parameters are the same as in Fig. 7. The cylindrical model has two cylindrical secondary dendrites, while the parabolic model has two parabolic secondary dendrites. Note that for each case the origin of the spatial coordinate is chosen to be in the branching node, placing the somatic node at the coordinate $x=-l_{0}$. (**b**) The normalised difference $\varDelta _{\mathrm{norm}}$ between the steady-state somatic responses of the two models

We investigated how the somatic responses at steady state $V_{\mathrm {ss}}(-l_{0},y)$ vary with the length of the primary dendritic segments $l_{0}$ (see Fig. 9a), using the Green’s functions obtained by the method of local point matching (the detailed calculations can be found in Appendix 4). A noticeable difference can be found locally on the secondary segments. The scale of the difference is modulated by $l_{0}$, which is mainly due to the signal loss along the primary segments. Introducing the normalised difference $\varDelta _{\mathrm {norm}}=V_{{\mathrm{ss}},p}(-l_{0},y)/V_{{\mathrm{ss}},c}(-l_{0},y)-1$, we can see from Fig. 9b that the overall trend of $\varDelta _{\mathrm{norm}}$ remains unchanged; $\varDelta _{\mathrm{norm}}>0$ for most of $y\in(0,l_{1}]$. In contrast, if the primary dendritic segment tapers but the secondary segments not, we observed $\varDelta _{\mathrm{norm}}>0$ for most of $y\in [-l_{0},0)$ instead. When both primary and secondary segments are tapering, $\varDelta _{\mathrm{norm}}>0$ can be observed for most of $y\in [-l_{0},l_{1}]$.

## Discussion

In this paper we present a unified approach for calculating the Green’s functions in a quasi-active (or passive) neuron with an arbitrary branching dendritic structures and tapering segments. It extends the sum-over-trips framework introduced for passive cylindrical dendrites [2] and quasi-active cylindrical dendrites [8], and generalises the work in [27] for single tapering cables. We also demonstrate how the solutions can be found in compact algebraic forms (instead of being represented as infinite sums) using the previously developed method of local point matching [43]. The obtained compact Green’s function solutions allow one to conduct mathematical analysis and efficient numerical simulations for better understanding the role of dendritic morphology on neuronal signal modulations. These solutions can be naturally reduced to the models with cylindrical dendritic structures to recover the results in [2, 8, 43] by considering $r(x)=r_{c}$. Note that, since the spatial scaling factor of the injected current has been incorporated in the form of the Green’s function in this paper (instead of being present in the function $I_{0}(x,t)$ as in previous studies [2, 8, 43]), the recovered Green’s functions for the cylindrical models differ from the previously obtained Green’s functions by a factor $Dr_{a}=1/(2\pi Cr_{c})$. The form of the Green’s function given here thus simplifies the reciprocity identity as

86 $$ G_{ij}(x,y)=G_{ji}(y,x), $$

by absorbing location dependent coefficients (a proof is given in Appendix 5).

Our comparison between the tapering and cylindrical models indicates that the parabolic geometry tends to increase somatic voltage responses driven by distal dendritic inputs and that this increase can be attributed to the local dendritic taper alone. The earlier work of Bird and Cuntz [4] mathematically justifies that the parabolic dendritic taper provides the optimal signal transfer from the input location to the soma in a model of a single tapering cable. Our study considered a still simplified, but nevertheless a more advanced ‘Y’-shaped tree model, which indicates that this optimal signal transfer property is also applicable to more complex dendritic branching structures.

As it stands this modelling framework excludes any active dynamics that can be attributed to the voltage-gated ionic channels. Mathematical analysis of the dendritic models with active properties is often impossible due to nonlinearities in the underlying equations, with a few exceptions of dendritic models with hot spots [28, 29] or simplified piecewise linear models of active spines used in frameworks such as the Spike–Diffuse–Spike (SDS) type model [7, 38]. It would be interesting to extend the SDS model from the cylindrical to tapering dendritic segments and investigate the geometric effects on the speed of wave propagation at the level of a single cable as well as on branching structures. Another possible extension of this work is to go beyond a single cell model to a network level. Considering a model of spatially extended neurons coupled by electrical synapses (gap-junctions) it is possible to extend the work in [39] and derive the node factors for gap-junctional boundary conditions located on tapering dendritic segments. The Green’s function for the whole network can then be efficiently computed using the method of local point matching [43]. Such extensions would allow us to rigorously investigate the effects of dendritic tapers on the voltage dynamics at the level of a single cell and electrically coupled neuronal networks with the dendritic and somatic membrane models being passive, quasi-active or active. Finally, the proposed framework is compatible with *stochastic cable theory* [40], allowing one to expand a recent work of Gowers et al. [13] of calculating the firing rates of branching neurons with tapering dendrites.

## Appendix 1: The generalised cable equation in a dendritic cable with a location-dependent radius

The classical cable theory introduces the cable equation with a constant dendritic radius $r_{c}$. Here we generalise the cable equation for the case of a continuously varying dendritic radius $r(x)$ to obtain the mathematical model given in (1). Similar derivations, for the passive membrane though, can be found in [4, 30].

Considering a small dendritic section $(x,x+\varDelta )$ and using the conservation of electrical currents we can write

87 $$ I_{c}(x)+I_{l}(x)+I_{\mathrm{ion}}(x)=I(x)+I_{\mathrm{in}}(x)-I(x+ \varDelta )-I_{\mathrm{in}}(x+\varDelta ), $$

where $I(x)$ and $I_{\mathrm{in}}(x)$ define the axial and the input currents respectively, and the capacitive, the leakage and the ion currents are described by the following equations:

88a $$\begin{aligned}& I_{c}(x)=C_{m}A_{s}(x,x+ \varDelta )\frac{\partial V}{\partial t}, \end{aligned}$$

88b $$\begin{aligned}& I_{l}(x)=g_{l}A_{s}(x,x+ \varDelta ) (V-E_{l}), \end{aligned}$$

88c $$\begin{aligned}& I_{\mathrm{ion}}(x)=g_{\mathrm{ion}}(V) A_{s}(x,x+ \varDelta ) (V-E_{\mathrm{ion}}). \end{aligned}$$

For simplicity we assume that the cross section of the cable is always a disc, and thus the membrane surface area of this section is

89 $$ A_{s}(x,x+\varDelta )= \int_{x}^{x+\varDelta }2\pi r(s)\sqrt{1+\bigl(r'(s) \bigr)^{2}}\,\textrm {d}s=2\pi \int_{x}^{x+\varDelta }\rho(s)\,\textrm {d}s. $$

Substituting Eqs. (88a), (88b) and (88c) into Eq. (87) and taking the limit $\varDelta \to0$, we obtain

90 $$ C_{m}\frac{\partial V}{\partial t}=-g_{l}(V-E_{l})-g_{\mathrm{ion}}(V) (V-E_{\mathrm{ion}})-\frac{1}{2\pi\rho(x)} \biggl[\frac{\partial I}{\partial x}+ \frac{\partial I_{\mathrm{in}}}{\partial x} \biggr]. $$

Without loss of generality we assume that an injected current $I_{\mathrm{inj}}(t)$ is applied to a small section $(x_{0},x_{0}+\varDelta )$. In the considered limit $\varDelta \to0$ it becomes an input current at a discrete point $x=x_{0}$, which gives

91 $$ \frac{\partial I_{\mathrm{in}}}{\partial x}\bigg|_{x \to x_{0}^{+}}=-I_{\mathrm{inj}}(t) \delta(x-x_{0}). $$

At the same time the axial current $I(x)$ is assumed to satisfy Ohm’s law, that is,

92 $$ V(x+\varDelta )-V(x)=-I(x)R(x), $$

where

93 $$ R(x)=\frac{R_{a}\varDelta ^{2}}{\int_{x}^{x+\varDelta }A_{c}(s)\,\textrm {d}s} $$

is the axial resistance of the section $(x,x+\varDelta )$, and

94 $$ A_{c}(x)=\pi r^{2}(x) $$

is the cross-sectional area. We can thus rewrite Eq. (92) and find the axial current as

95 $$ I(x)=-\frac{V(x+\varDelta )-V(x)}{\varDelta }\frac{\int_{x}^{x+\varDelta }A_{c}(s)\,\textrm {d}s}{R_{a}\varDelta }, $$

which reduces to

96 $$ I(x)=-\frac{A_{c}(x)}{R_{a}}\frac{\partial V}{\partial x}\quad\text{as } \varDelta \to0. $$

This leads to

97 $$ \frac{\partial I}{\partial x}=-\frac{\pi}{R_{a}}\frac {\partial}{\partial x} \biggl[r^{2}(x)\frac{\partial V}{\partial x} \biggr]. $$

Substituting Eqs. (91) and (97) into Eq. (90) we obtain

98 $$ \begin{aligned}[b] C_{m}\frac{\partial V}{\partial t}&=-g_{l}(V-E_{l})-g_{\mathrm{ion}}(V) (V-E_{\mathrm{ion}})\\&\quad+\frac{1}{2R_{a} \rho(x)}\frac{\partial}{\partial x} \biggl[r^{2}(x) \frac{\partial V}{\partial x} \biggr]+\frac{I_{{\mathrm{inj}}}(t)\delta (x-x_{0})}{2\pi\rho(x)},\end{aligned} $$

which is the generalised cable equation given in (1).

## Appendix 2: The Green’s function of the Helmholtz equation

The Green’s function of an inhomogeneous Helmholtz equation can be found as a solution to the following canonical form of the Helmholtz equation:

99 $$ \bigl[\nabla^{2} + k^{2} \bigr] G_{H}(X) = -\delta(X), \quad X\in\mathbb {R}, $$

which is known to be

100 $$ G_{H}(X) = \frac{i\exp(ik \vert X \vert )}{2k}, $$

where $k\in\mathbb {C}$ is the wavenumber and $i=\sqrt{-1}$ is the imaginary unit. In the special case of wavenumber $k=i\gamma(\omega)$ as in Eq. (20)

101 $$ G_{H}(X,\omega) = \frac{\exp(-\gamma(\omega) \vert X \vert )}{2\gamma (\omega)}. $$

Although the spatial normalisation mapping $\gamma:X\to\bar{x}$ makes the spatial coordinate a complex number, given any *ω* applying *γ* to Eq. (99) with $k=i\gamma(\omega)$, we obtain

102 $$ \bigl[\nabla^{2} + i^{2} \bigr] H_{\infty}(\bar{x}) = -\delta(\bar{x}), $$

where

103 $$ H_{\infty}(\bar{x}) = \frac{1}{2}\exp\bigl(-\gamma(\omega) \vert X \vert \bigr) = \frac{1}{2}\exp\biggl(-\bar{x}\operatorname {sgn}\biggl[ \frac{\bar{x}}{\gamma(\omega)} \biggr] \biggr), $$

which is identical to Eq. (24).

## Appendix 3: The new node factors in the extended sum-over-trips framework for tapering dendrites

Here we derive the node factors listed in Sect. 3.5. Since a node factor at any node depends solely on the boundary conditions imposed by this node (proved in [2]), we consider a single node of each type and assume that only semi-infinite dendritic cables are attached to this considered node. As a starting point we rewrite Eq. (47) as

104 $$ G_{ij}(x,y)=\eta_{j}(y)\phi_{i}(X) \sum_{\mathrm{trip}}A_{\mathrm{trip}}(\omega)H_{\infty}\bigl(\bar{l}_{\mathrm{trip}}({x},{y})\bigr), $$

where

105 $$ \eta_{j}(y)=\frac{1}{z_{j}(y)\phi_{j}(Y)}. $$

To simplify the notation, we omit *ω* in all the functions here and we work simultaneously with the two spatial coordinates linked by the bijective mapping $\mu:x\to X$. Without loss of generality we also assume that a node is located at $x=0$ (and thus $X=0$) in all three types of nodes, imposing the boundary conditions listed in Sect. 3.3 on the Green’s function (104). In practice any node factor should be evaluated with its local parameters, and its value is independent of the choice of local spatical coordinates.

### 3.1 Terminal node

Under the above assumptions the dendritic morphology is simply a semi-infinite cable. Applying the sum-over-trips framework there are only two trips, a direct trip and a reflective one. Equation (104) thus reduces to

106 $$ G(x,y)=\eta(y)\phi(X) \bigl[H_{\infty}(\gamma Y-\gamma X)+ \alpha_{k} H_{\infty}(\gamma Y+\gamma X) \bigr], $$

where $\alpha_{k}$, $k\in\{o,c\}$ is the reflective node factor for killed and sealed terminals respectively. If the terminal node is killed, the boundary condition (40) is imposed, equivalently,

107 $$ G(0,y)=0. $$

Substituting the Green’s function (106) into Eq. (107) we obtain

108 $$ \eta(y)\phi(0) (1+\alpha_{o})H_{\infty}(\gamma Y)=0, $$

which simply gives

109 $$ \alpha_{o}+1=0 $$

as all the other functions on the left hand side are positive.

If the terminal node is sealed, the boundary condition (41) is imposed, which gives

110 $$ \frac{\partial G}{\partial x}\bigg|_{(0,y)}=0. $$

By the chain rule

111 $$ \frac{\partial G}{\partial x}\bigg|_{(0,y)}=\frac{\textrm {d}X}{\textrm {d}x}\frac {\partial G}{\partial X}\bigg|_{(0,Y)}= \frac{1}{\lambda(0)}\frac{\partial G}{\partial X}\bigg|_{(0,Y)}, $$

which implies

112 $$ \frac{\partial G}{\partial X}\bigg|_{(0,Y)}=0. $$

The following identity is worth noting:

113 $$ \frac{\partial}{\partial X} H_{\infty}(\gamma X)=-\gamma H_{\infty}(\gamma X), \quad x\geq0, $$

which can be easily deduced from the definition (24) of $H_{\infty}(\bar{x})$. Therefore,

114 $$ \frac{\partial G}{\partial X}=\eta(y)\phi(X) \biggl( \biggl[\gamma-\frac{1}{2} \xi(X) \biggr]H_{\infty}(\gamma Y-\gamma X)-\alpha_{c} \biggl[ \gamma+\frac{1}{2}\xi(X) \biggr] H_{\infty}(\gamma Y+\gamma X) \biggr), $$

which gives

115 $$ \frac{\partial G}{\partial X}\bigg|_{(0,Y)}=\eta(y)\phi(0) \biggl( \biggl[\gamma- \frac{1}{2}\xi(0) \biggr]-\alpha_{c} \biggl[\gamma+ \frac{1}{2}\xi(0) \biggr] \biggr) H_{\infty}(\gamma Y). $$

Substituting it into Eq. (112) gives

116 $$ \alpha_{c}=\frac{\gamma-\xi(0)/2}{\gamma+\xi(0)/2}. $$

It can be easily checked that the node factor (109) is identical to (54), and (116) is identical to (53).

### 3.2 Branching node

Here we consider a dendritic morphology consisting of *K* semi-infinite dendritic cables attached to a branching node, and without loss of generality the input location *y* is assumed to be on cable 1. We define $\alpha_{k}=\alpha_{k1}$, $k\in\{1,2,3,\ldots,K\}$ to be the node factors for the trip travelling from segment *k* to 1, and consider two cases: the output is located (i) on cable 1 and (ii) on cable $k\neq1$. In these two cases the sum-over-trips framework reduces Eq. (104) to

117a $$\begin{aligned}& G_{1}(x_{1},y)=\eta_{1}(y) \phi_{1}(X) \bigl[H_{\infty}(\gamma_{1} Y- \gamma_{1} X)+\alpha_{1} H_{\infty}( \gamma_{1} Y+\gamma_{1} X) \bigr], \end{aligned}$$

117b $$\begin{aligned}& G_{k}(x_{k},y)=\eta_{1}(y) \phi_{k}(X)\alpha_{k} H_{\infty}( \gamma_{1} Y+\gamma_{k} X),\quad\text{for $k\neq1$}. \end{aligned}$$

Requiring the continuity of membrane potentials (42a), i.e.

118 $$ G_{1}(0,y)=G_{k}(0,y), $$

and using the Green’s functions in (117a) and (117b), we obtain

119 $$ \phi_{1}(0) (1+\alpha_{1})= \phi_{k}(0)\alpha_{k}, \quad k\neq1. $$

The conservation of electrical currents (42b) must also be satisfied, that is,

120 $$ 0=\sum_{k=1}^{K} \frac{1}{r_{a,k}(0)}\frac{\partial G_{k}}{\partial x}\bigg|_{(0,y)}=\sum _{k=1}^{K}\frac{1}{r_{a,k}(0)\lambda_{k}(0)}\frac{\partial G_{k}}{\partial X}\bigg|_{(0,Y)}, $$

where

121a $$\begin{aligned}& \frac{\partial G_{1}}{\partial X}\bigg|_{(0,Y)}=\eta_{1}(y) \phi_{1}(0) \biggl( \biggl[\gamma_{1}-\frac{1}{2} \xi_{1}(0) \biggr]-\alpha_{1} \biggl[\gamma_{1}+ \frac{1}{2}\xi_{1}(0) \biggr] \biggr)H_{\infty}( \gamma_{1} Y), \end{aligned}$$

121b $$\begin{aligned}& \frac{\partial G_{k}}{\partial X}\bigg|_{(0,Y)}=\eta_{1}(y) \phi_{k}(0) \biggl(-\alpha_{k} \biggl[\gamma_{k}+ \frac{1}{2}\xi_{k}(0) \biggr] \biggr) H_{\infty}( \gamma_{1} Y). \end{aligned}$$

By substitution and rearrangement, Eq. (120) can be reduced to

122 $$ 0=2z_{1}(0)-(\alpha_{1}+1)\sum _{k=1}^{K} z^{*}_{k}(0), $$

where $z_{k}(0)$ and $z_{k}^{*}(0)$ are defined in Eqs. (50) and (56) respectively. Combining Eqs. (119) and (122), we can solve for $\alpha_{1}$ first and then find $\alpha_{k}$ for $k\neq1$ as follows:

123a $$\begin{aligned}& \alpha_{1}=\frac{2z_{1}(0)}{\sum_{k=1}^{K} z_{k}^{*}(0)}-1, \end{aligned}$$

123b $$\begin{aligned}& \alpha_{k\neq1}=\frac{\phi_{1}(0)}{\phi_{k}(0)}\frac{2z_{1}(0)}{\sum _{k=1}^{K} z_{k}^{*}(0)}, \end{aligned}$$

giving us the node factors (57) and (58).

### 3.3 Somatic node

Here we consider a dendritic morphology that is identical to that in the previous section, and thus we can use exactly the same Green’s functions (117a) and (117b). However, the node in this section is a soma, and therefore the boundary conditions (43a), (43b) and (43c) are imposed. Performing the Laplace transform on Eqs. (43b) and (43c), we obtain in the frequency domain

124 $$ z_{S} V_{S}=\sum _{k=1}^{K}\frac{1}{r_{a,k}(0)}\frac{\partial V_{k}}{\partial x}\bigg|_{x=0}, $$

where $z_{S}$ is defined in Eq. (64). In addition, the continuity of membrane potentials (43a) implies the same condition as (118):

125 $$ G_{1}(0,y)=G_{k}(0,y)=G_{S}(y), $$

where $G_{S}(y)$ is the somatic Green’s function satisfying Eq. (124). Therefore this equation can be equivalently rewritten in terms of the Green’s functions as

126 $$ z_{S}G_{1}(0,y)=\sum _{k=1}^{K}\frac{1}{\lambda_{k}(0)r_{a,k}(0)}\frac{\partial G_{k}}{\partial X}\bigg|_{(0,Y)}, $$

the left-hand side of which can be found by the direct substitution as

127 $$ z_{S}\eta_{1}(y)\phi_{1}(0) (1+ \alpha_{1})H_{\infty}(\gamma_{1} Y), $$

and the right-hand side can be reduced to

128 $$ \eta_{1}(y)\phi_{1}(0)H_{\infty}( \gamma_{1} Y) \biggl[\frac{2\gamma_{1}}{\lambda_{1}(0)r_{1}(0)}-(\alpha_{1}+1)\sum _{k}\frac{\gamma_{k}+\xi_{k}(0)/2}{\lambda_{k}(0)r_{k}(0)} \biggr] $$

by using the derivatives previously found in Eqs. (121a) and (121b). Thus Eq. (126) is reduced to

129 $$ z_{S}(1+\alpha_{1})=2z_{1}(0)-( \alpha_{1}+1)\sum_{k=1}^{K} z_{k}^{*}(0), $$

which gives

130a $$\begin{aligned}& \alpha_{1}=\frac{2z_{1}(0)}{z_{S}+\sum_{k=1}^{K} z_{k}^{*}(0)}-1, \end{aligned}$$

130b $$\begin{aligned}& \alpha_{k\neq1}=\frac{\phi_{1}(0)}{\phi_{k}(0)}\frac{2z_{1}(0)}{z_{S}+\sum _{k=1}^{K} z_{k}^{*}(0)}. \end{aligned}$$

It can be easily proved that the above expressions are identical to the node factors (61) and (62).

## Appendix 4: The Green’s function in the ‘Y’-shaped dendritic tree

To find the Green’s function for the ‘Y’-shaped dendritic model discussed in Sect. 4.4, we apply the sum-over-trips framework and use the method of local point matching [43]. We denote the dendritic segments by 0 (primary) and 1 (secondary), and the nodes by *S* (somatic), *B* (branching) and *T* (sealed terminal), and thereby identify all node factors below.

- At the somatic node,
  131 $$ \alpha^{S}_{00}=2p^{S}_{0}-1, $$
  where
  132 $$ p^{S}_{0}=\frac{z_{0}^{S}}{z_{0}^{S}-\kappa_{0}+z_{S}}. $$
- At the branching node,
  133a $$\begin{aligned}& \alpha^{B}_{00}=2p^{B}_{0}-1, \end{aligned}$$
  133b $$\begin{aligned}& \alpha^{B}_{11}=\alpha^{B}_{22}=2p^{B}_{1}-1, \end{aligned}$$
  133c $$\begin{aligned}& \alpha^{B}_{01}=\alpha^{B}_{02}=2p^{B}_{1} \varPhi_{01}, \end{aligned}$$
  133d $$\begin{aligned}& \alpha^{B}_{10}=\alpha^{B}_{20}=2p^{B}_{0} \varPhi_{10}, \end{aligned}$$
  133e $$\begin{aligned}& \alpha^{B}_{21}=\alpha^{B}_{12}=2p^{B}_{1}, \end{aligned}$$
  where
  134a $$\begin{aligned}& p_{0}^{B}=\frac{z_{0}^{B}}{(z_{0}^{B}+\kappa_{0})+2(z_{1}^{B}-\kappa_{1})}, \end{aligned}$$
  134b $$\begin{aligned}& p_{1}^{B}=\frac{z_{1}^{B}}{(z_{0}^{B}+\kappa_{0})+2(z_{1}^{B}-\kappa_{1})}, \end{aligned}$$
  and
  135 $$ \varPhi_{10}=\varPhi_{01}^{-1}= \frac{\phi_{0}^{B}}{\phi_{1}^{B}}. $$
- At the two sealed terminal nodes,
  136 $$ \alpha_{11}^{T}=\frac{2z_{1}^{T}}{z_{1}^{T}+\kappa_{1}}-1. $$

All the variables should be evaluated according to the definitions in Sect. 3.5. The details are omitted here. Assuming that the output is located at the soma and applying the method of local point matching we obtain

137a $$\begin{aligned}& J_{a}=J_{b} h(L_{0}) \alpha_{00}^{S} + h(0) \alpha_{00}^{S}, \end{aligned}$$

137b $$\begin{aligned}& J_{b}=J_{a} h(L_{0}) \alpha_{00}^{B}+ h(L_{0}) \alpha_{00}^{B}+ J_{d} h(L_{1}) \alpha_{10}^{B}+ J_{e} h(L_{2}) \alpha_{20}^{B}, \end{aligned}$$

137c $$\begin{aligned}& J_{c}=J_{a} h(L_{0}) \alpha_{01}^{B}+ h(L_{0}) \alpha_{01}^{B}+ J_{d} h(L_{1}) \alpha_{11}^{B}+ J_{f} h(L_{2}) \alpha_{21}^{B}, \end{aligned}$$

137d $$\begin{aligned}& J_{d}=J_{c} h(L_{1}) \alpha_{11}^{T}. \end{aligned}$$

Expressions for $J_{e}$, $J_{f}$ are omitted here since the two secondary dendritic segments are identical. Equivalently, the system (137a), (137b), (137c) and (137d) can be rewritten in the following matrix form:

138 $$ \begin{bmatrix} -1 & h(L_{0}) \alpha_{00}^{S} & 0 & 0 \\ h(L_{0}) \alpha_{00}^{B} & -1 & 0 & 2h(L_{1}) \alpha_{10}^{B} \\ h(L_{0}) \alpha_{01}^{B} & 0 & -1 & h(L_{1}) [\alpha_{11}^{B}+\alpha_{21}^{B}] \\ 0 & 0 & h(L_{1}) \alpha_{11}^{T} & -1 \end{bmatrix} \begin{bmatrix} J_{a}+1 \\ J_{b} \\ J_{c} \\ J_{d} \end{bmatrix} = \begin{bmatrix} -(\alpha_{00}^{S}+1) \\ 0 \\ 0 \\ 0 \end{bmatrix} , $$

where $L_{0}=\gamma_{0} \mu_{0}(l_{0})$, $L_{1}=\gamma_{1} \mu_{1}(l_{1})$, and solved for the unknown *J* functions. This allows us to compute $J_{y}$ as

139 $$ J_{y}= \textstyle\begin{cases} (J_{a}+1) h(L_{y})+J_{b} h(L_{0}-L_{y}), &\text{for } 0\leq y\leq l_{0},\\ J_{c} h(L_{y-l_{0}})+J_{d} h(L_{1}-L_{y-l_{0}}), &\text{for } l_{0}< y\leq l_{0}+l_{1}, \end{cases} $$

where $L_{y}=\gamma_{0} \mu_{0}(y)$, $L_{y-l_{0}}=\gamma_{1} \mu_{1}(y-l_{0})$, and construct the Green’s function using Eq. (65).

## Appendix 5: The reciprocity identity for an arbitrary dendritic tree

Equation (18) of the model in the Laplace domain is the second-order linear ordinary differential equation and can thus be rewritten in the Sturm–Liouville form. The differential operator is self-adjoint, and therefore the Green’s function must be symmetric in space [36], supporting the reciprocity principle $G(x,y)=G(y,x)$ for the Green’s function in an infinite single cable. Here we demonstrate that this principle is also valid on an arbitrary dendritic tree with any boundary conditions discussed in Sect. 3.3. For any trip and its reversal configuration, namely ‘pirt’, we can write down the trip coefficients and find the ratio between them as

140 $$ \frac{A_{\mathrm{trip}}}{A_{\mathrm {pirt}}}=\frac{\alpha_{ik_{1}}\alpha_{k_{1}k_{2}}\alpha_{k_{2}k_{3}}\cdots\alpha _{k_{n-1}k_{n}}\alpha_{k_{n}j}}{\alpha_{jk_{n}}\alpha_{k_{n}k_{n-1}}\alpha _{k_{n-1}k_{n-2}}\cdots\alpha_{k_{2}k_{1}}\alpha_{k_{1}i}}, $$

given that all reflective node factors have cancelled each other. Assuming $k_{0}=i$, $k_{n+1}=j$, and segments $k_{m}$ and $k_{m+1}$ are connected at node $\chi_{m}$ for all $m\in\{0,1,2,\dots,n\}$, using either Eq. (58) or (62) we have

141 $$ \frac{\alpha_{k_{m}k_{m+1}}}{\alpha_{k_{m+1}k_{m}}}=\frac {z_{k_{m+1}}(\chi_{m})}{z_{k_{m}}(\chi_{m})} \bigl[ \varPhi^{(\chi_{m})}_{k_{m}k_{m+1}} \bigr]^{2}=\frac{\gamma_{k_{m+1}}}{\gamma_{k_{m}}}. $$

In addition, any given trip and its reversal trip are identical in their lengths, regardless of any scaling (i.e. $\bar{l}_{\mathrm {trip}}=\bar{l}_{\mathrm{pirt}}$), which gives

142 $$ \frac{\sum A_{\mathrm{trip}}H_{\infty}(\bar{l}_{\mathrm{trip}})}{\sum A_{\mathrm{pirt}}H_{\infty}(\bar{l}_{\mathrm{pirt}})}=\frac{\gamma _{j}}{\gamma_{i}}. $$

Therefore using Eq. (47) we obtain

143 $$ \frac{G_{ij}(x,y)}{G_{ji}(y,x)}=\frac{z_{i}(x)}{z_{j}(y)} \bigl[ \varPhi_{ji}(y,x) \bigr]^{2}\frac{\gamma_{j}}{\gamma_{i}}=1, $$

which gives the reciprocity identity (86) either in the frequency or time domain.

There is an immediate corollary from the reciprocity identity (86) stating that $G_{ij}(x,y)$ is continuous with respect to the input location *y*. To prove this we recall the continuity of membrane potentials at the nodes discussed in Sect. 3.3, which ensures that $G_{ji}(y,x)$ is continuous in *y*, and thus $G_{ij}(x,y)=G_{ji}(y,x)$ is also continuous in *y*. Although the corollary seems to be trivial, its important consequence is the case of having the input location *y* at a node (e.g. the soma). In such a scenario we can place the input at *y* on any of the dendritic segments that are attached to that node and then consider the distance between *y* and the node to be zero. The corollary guarantees the uniqueness of the solution, even if we choose different attached segments in such circumstances. Therefore the framework can be applied directly to any input location.
